# The Intervention and Mechanism of Action for Aloin against Subchronic Aflatoxin B1 Induced Hepatic Injury in Rats

**DOI:** 10.3390/ijms222111620

**Published:** 2021-10-27

**Authors:** Hanyi Hua, Jie Sheng, Yan Cui, Wenyi Zhang, Bin Hu, Yuliang Cheng, Yahui Guo, He Qian

**Affiliations:** 1 School of Food Science and Technology, Jiangnan University, Wuxi 214122, China; huabing1106@126.com (H.H.); shengjie970704@163.com (J.S.); nanzwy@163.com (W.Z.); wxfoodcyl@126.com (Y.C.); guoyahui@jiangnan.edu.cn (Y.G.); 2 School of Biotechnology, Jiangnan University, Wuxi 214122, China; hubinwx@163.com; 3 Institute of Agricultural Products Processing, Ningbo Academy of Agricultural Sciences, Ningbo 315040, China

**Keywords:** aflatoxins, AFB1, CYP1A2, CYP3A4, aloin

## Abstract

As a class of difurancoumarin compounds with similar structures, aflatoxins (AF) are commonly found in the environment, soil, and food crops. AF pose a serious threat to the health of humans, poultry, and livestock. This study aimed to investigate the neuroprotective effect and detailed mechanism of aloin on hepatic injury induced by subchronic AFB1 in rats. The result showed that aloin could significantly inhibit the decrease in food intake, body weight growth, immune organ index, and serum albumin content caused by long-term AFB1 exposure. Meanwhile, aloin reduced the level of serum liver function and improved renal swelling and pathological changes of liver tissue. Aloin could also inhibit liver lipid peroxidation and improve liver antioxidant capacity. Further investigation revealed that aloin inhibited the activity and expression of hepatic CYP1A2 and CYP3A4 and down-regulated *IL-1β* expression in subchronic AFB1-induced liver injury rats. The above study demonstrated that aloin played an important role in blocking or delaying the development process of subchronic AFB1-induced hepatotoxicity. Therefore, aloin is considered to have a potential role as a protective agent against AFB1.

## 1. Introduction

Aflatoxins (AF) are a class of difurancoumarin compounds with similar structures that are mainly produced by the metabolism of toxic Aspergillus parasitic and Aspergillus flavus [1]. From the chemical structure perspective, AF structures contain 1,2-benzopyrone and bisfuran rings. The former can enhance the toxicity of the latter. Currently, the most common AF are AFB1, AFB2, AFG1, AFG2, AFM1, and AFM2. The structures are shown in the Supplementary Materials (Figure S1). The first four are usually natural, meanwhile the last two are formed by metabolic hydroxylation of AFB1 and AFB1 in vivo. Among all, AFB1 is the main existing form and has the highest toxicity ever. It was designated as a human class I carcinogen by the International Agency for Research on Cancer (IARC) as early as 1993 [2,3,4]. AFB1 is commonly found in the environment, soil, and food crops, and this seriously threatens the health of humans, poultry, and livestock.

AFB1 is highly toxic to the liver and can cause hepatocyte necrosis, bile duct hyperplasia, liver fibrosis, and ultimately liver cancer as it represents one of the main causes of the development of primary hepatocellular carcinoma [5,6]. As a precancerous substance, AFB1 toxicity is mediated by the phase I metabolic enzyme cytochrome P450 enzyme system (CYP450). After being activated by CYP450, part of AFB1 is O-dealkylated or hydroxylated to AFP1, AFM1, or AFQ1 and excreted with feces and urine; the other part is activated to generate final carcinogens AFB-8,9-epoxide (AFBO) under the epoxidation of cytochromes P4501A2 and 3A4 (CYP1A2 and CYP3A4) [7,8]. AFBO is the primary highly active electrophilic compound that mediates liver toxicity induced by AFB1; part of it is detoxified by combining with reduced glutathione (GSH) under the catalysis of glutathione S-transferase (GST) [9,10]. The undetoxified AFBO, due to its high electronic affinity, can bind to the N7 position of DNA guanine to form AFB1-N7-Gua conjugates, which subsequently triggers a variety of DNA damage modes. This process is the origin of AFB1-induced gene mutation and the first step of inducing liver cancer [11]. Studies have pointed out that AFBO can induce a point mutation in the third base of codon 249 of the tumor suppressor gene p53, mutating the codon AGG to AGT, which will lead to changes in the encoded amino acids and the occurrence of liver cancer [7]. AFBO can covalently combine with lysine groups of albumin in serum to form AFB1-albumin adduct, which will not be repaired and would therefore remain in the blood to produce toxicity [12].

Aloin, as a derivative of aloe-emodin C-glucoside, is one of the commonly used active ingredients in aloe exudate, which accounts for 15–40% of the exudate [13]. Its structure is shown in the Supplementary Materials (Figure S2). Aloin has an excellent laxative effect and has been widely used in constipation treatment drugs. Recent studies have found that aloin also plays a vital anti-oxidative and anti-inflammatory role. Asamenew et al. found that aloin has potent free radical scavenging ability and can effectively scavenge hydroxyl radicals and 2,2-diphenyl-1-picrylhydrazyl (DPPH) free radicals in vitro antioxidant experiments [14]. Meanwhile, aloin exerts a powerful inhibitory effect on hepatic retinopathy. Eunsoo et al. evaluated the effects of aloin on Müller cell dysfunction in a rat model of thioacetamide (TAA)-induced hepatic retinopathy. Administration of aloin suppresses liver injury as well as Müller cell swelling through the normalization of Kir4.1 and aquaporin-4 channels, which indicates that aloin may be helpful to protect retinal injury associated with liver failure [15]. Additionally, aloin can also improve the colonic oxidative stress state of the colonic injury model in rats and significantly inhibit the production of MDA while improving the levels of a series of antioxidants such as GSH-PX, GSH, and catalase (CAT) in the colon [16]. Inflammation is a natural reaction of the body to injury, mainly manifested by redness, fever, pain, and functional loss, which can hinder the healing of injury [17]. Studies have shown that aloin also has prominent anti-inflammatory effects. By feeding Wild-type or nuclear erythroid 2-related factor 2 (Nrf2) knock-out mice with the choline-deficient, l-amino acid-defined, high-fat diet, researchers found that aloin supplementation possesses antioxidant, anti-inflammatory, and anti-apoptotic activity effects on non-alcoholic steatohepatitis mice by activating the Nrf2/HO-1 pathway [18]. Park et al. [19] studied the effect of aloin on the LPS-induced inflammatory response in mouse macrophages using the LPS-induced inflammatory response as an experimental model, and the results showed that aloin reduces the production of lipopolysaccharide (LPS)-induced NO and strongly inhibits the expression of the inducible nitric oxide synthase (iNOS) gene, thus reducing the inflammatory response of cells. Subsequently, the rat colon inflammation induced by dextran sulfate sodium was used as a model in in vivo tests, and the effect of dietary intake of aloin on inflammation was investigated. The results showed that aloin exerts prominent relief effects on colon inflammation, which reduces the content of leukotriene B4 in plasma, the activity of myeloperoxidase in the colon, and inhibits the expression of *TNF-α* and *IL-1β* mRNA in the colon [20].

At present, the research on the efficacy of aloe mainly focuses on wound healing, immune regulation, anti-tumor, and radiation resistance. Although there have been reports that aloe and its extracts have potential liver-protective effects, little research has been performed on its specific mechanism of action and functional components. Our previous laboratory studies have found that aloin intervention can reduce the degree of acute and chronic alcoholic liver injury, and has significant antioxidant, anti-inflammatory, and lipid metabolism-regulating effects, which provides a specific theoretical basis for the chemical prevention of AFB1 toxicity. The purpose of this study is to reveal the role and mechanism of aloin in the development of liver injury induced by AFB1, to provide new insights for the development of chemical preventive measures against hepatotoxicity of AFB1, and to provide a firm theoretical basis for the comprehensive development and utilization of aloe.

## 2. Results

### 2.1. Growth Status and Organ Index of Mice

**Figure 1:**
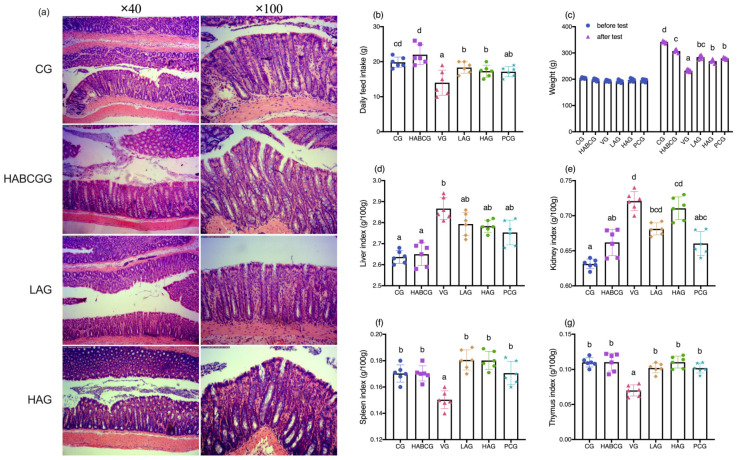
Effect of aloin supplementation on colon tissue (**a**), the average daily food intake of rats fed AFB1 (**b**), the body weight (**c**) and viscera index (**d**–**g**) in AFB1-treated rats. CG, control group; HABCG, high-dose aloin blank control group; VG, vehicle group; LAG, HAG, low- and high-dose aloin intervention group; PCG, positive control group (values with different letters represent statistical significance difference among groups, *p* < 0.05).

We discussed whether the dosage of aloin will bring side effects to the intestinal tract of the body before exploring its protective effect on hepatic injury. The observation results showed (in Figure 1a) that no abnormal structure was found in the colon of each group of rats, the glands were arranged orderly, the shape and size of goblet cells were standard, and there were no pathological changes such as inflammatory infiltration. Therefore, the aloin used in the experiment was safe in dosage and did not damage the colon of rats.

Studies have shown that prolonged exposure to AFB1 can lead to chronic poisoning, causing the reduction of digestive tract enzymes such as pancreatic lipase, trypsin, and amylase, resulting in the malabsorption of nutrients and metabolic disorders in the body, loss of appetite, and consequently causing the slow weight gain [21]. The long-term exposure of AFB1 resulted in a significant decrease in the average of daily food intake of rats, as shown in Figure 1b. However, aloin can effectively relieve anorexia caused by AFB1, and its daily average food intake was significantly higher than that of the vehicle group (*p* < 0.05). Additionally, the average of daily food intake of high-dose aloin alone did not reach an obvious difference compared with the average level of the control group, indicating that aloin had no significant effect on the average of daily food intake of normal rats.

To assess the effect of aloin on the body weight of rats, we tracked the body weight of rats in each treatment group. As shown in Figure 1c, the weight of rats in the vehicle group was lower than that in the control group after a continuous intake of AFB1 for 5 weeks (*p* < 0.001). However, it is interesting to note that the slow weight growth caused by AFB1 could be effectively alleviated by intragastric administration of aloin. Compared with the vehicle group, the weights of the low-dose and high-dose groups were increased (*p* < 0.01). Additionally, the final body weight of the aloin group was lower than the average level of the control group when high-dose aloin was given alone, which may be related to the functional effects of aloin in reducing weight and relaxing bowels. Compared with the control group, the continuous intake of AFB1 for five weeks increased the liver index (*p* < 0.05, Figure 1d) and kidney index (*p* < 0.001, Figure 1e) of the rats and decreased the spleen index (*p* < 0.05, Figure 1f) and thymus index (*p* < 0.001, Figure 1g) at the same time, indicating that AFB1 damaged the liver and kidney as well as the immune system of the body. The result showed that liver and kidney swelling caused by AFB1 was inhibited to a certain extent under the intervention of aloin, but the difference was not significant compared with the vehicle group (*p* > 0.05). Interestingly, aloin could inhibit the atrophy of immune organs caused by AFB1, the spleen index and thymus index in high- and low-dose groups were higher than those in the vehicle group (*p* < 0.05), and the level was equivalent to that in control group. It is presumed that those phenomenon are related to the anti-inflammatory effect of aloin, and the specific mechanism needs further study.

### 2.2. Changes in Liver Tissue and Serum Liver Function

**Figure 2:**
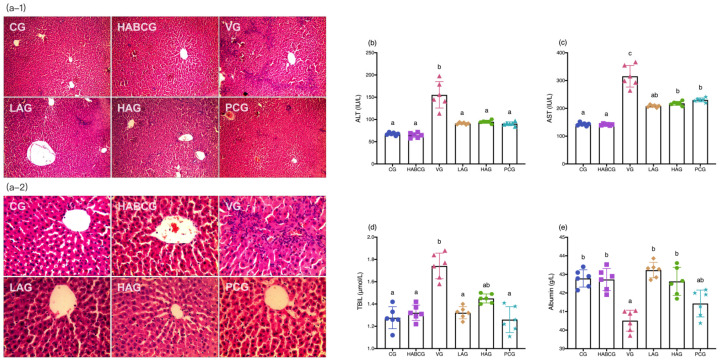
Effect of aloin supplementation on subchronic AFB1-induced liver damage in rats. (**a–1**) magnification 100×, (**a–2**) magnification 400×. The liver function indexes were shown in (**b**–**e**). CG, control group; HABCG, high-dose aloin blank control group; VG, vehicle group; LAG, HAG, low- and high-dose aloin intervention group; PCG, positive control group; ALT, alanine aminotransferase; AST, aspartate transaminase; TBIL, total bilirubin (values with different letters represent statistical significance difference among groups, *p* < 0.05).

The H&E staining pathology of the liver tissue of rats in each treatment group was shown in Figure 2a–1,a–2. As shown in the figure, the liver lobules of the control group rats were good in shape, the liver cords were arranged in a radial pattern and were orderly arranged, and the liver cells were complete, uniform in size, and uniform in the cytoplasm. In the vehicle group, pathological changes occurred in the liver tissue, with the abnormal structure of hepatic lobules, disordered arrangement of hepatic cords, swelling and uneven size of hepatocytes, visible binuclear, severe infiltration of inflammatory cells in portal area, and occasional inflammation near the central portal vein. Compared with the vehicle group, both the aloin intervention group and the positive group showed different degrees of pathological changes. However, the degree of liver cell swelling was decreased, the structure of liver lobules was healthy, and the inflammatory infiltration was also significantly inhibited, which indicated that aloin also played a functional role in protecting liver from subchronic injury caused by AFB1. Additionally, compared with the control group, the liver tissue morphology of the rats in the high-dose aloin blank control group alone was not abnormal, and the dose used in the experiment was safe and non-toxic.

In order to evaluate the intervention effect of aloin on the subchronic liver injury model of AFB1, the liver function indexes such as ALT (Figure 2b), AST (Figure 2c), TBIL (Figure 2d), and albumin (Figure 2e) in the serum of rats were detected. It can be seen from the data that the administration of high-dose aloin did not affect various liver function indexes, indicating that the aloin dose used in the experiment had no side effect on the liver, and the dose was reasonable. Compared with the control group, ALT (*p* < 0.01), AST (*p* < 0.001), and TBIL (*p* < 0.05) in the vehicle group were abnormally increased, while the albumin content that represented the hepatic synthetic function had decreased significantly (*p* < 0.05) following long-term AFB1 intake. Aloin intervention effectively reduced ALT, AST, and TBIL levels, which were significantly lower than those of vehicle group (*p* < 0.05), and the ALT level was close to that of the control group. The serum albumin content was higher than that of the vehicle group (*p* < 0.05), which was the same as the level of the control group. However, the dose had no significant effect on the reduction of the above serum indexes; this may be related to the lower bioavailability of aloin.

### 2.3. Effect of Aloin on Serum Liver Function, Liver Antioxidant Capacity, and Phase II Enzyme GST

**Figure 3:**
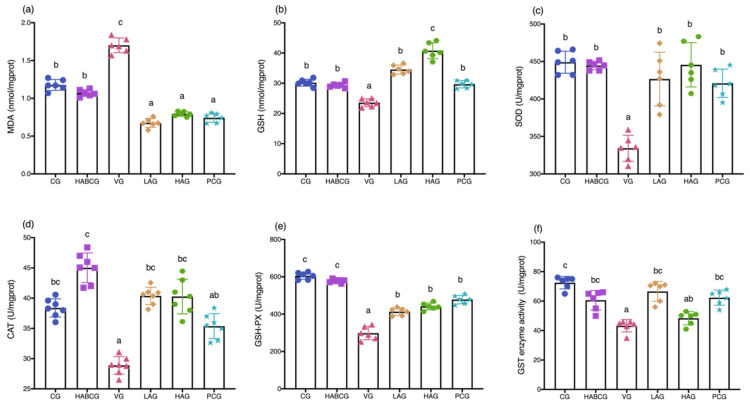
Effect of aloin supplementation on lipid peroxidation (**a**), antioxidant status (**b**–**e**), and hepatic GST (**f**) in AFB1-treated rats. CG, control group; HABCG, high-dose aloin blank control group; VG, vehicle group; LAG, HAG, low- and high-dose aloin intervention group; PCG, positive control group; MDA, malondialdehyde; GSH, reduced glutathione; SOD, superoxide dismutase; CAT, catalase; GSH-Px, glutathione peroxidase; GST, glutathione-S-transferases (values with different letters represent statistical significance difference among groups, *p* < 0.05).

Compared with the control group, the level of MDA, a lipid peroxidation marker in the liver, was increased in the vehicle group (*p* < 0.05, Figure 3a). Compared with the vehicle group, lipid peroxidation induced by AFB1 was effectively relieved under the intervention of aloin. MDA level in the high- and low-dose group was significantly reduced, even lower than the normal level, which may be closely related to the effective free radical scavenging ability of aloin.

**Figure 4:**
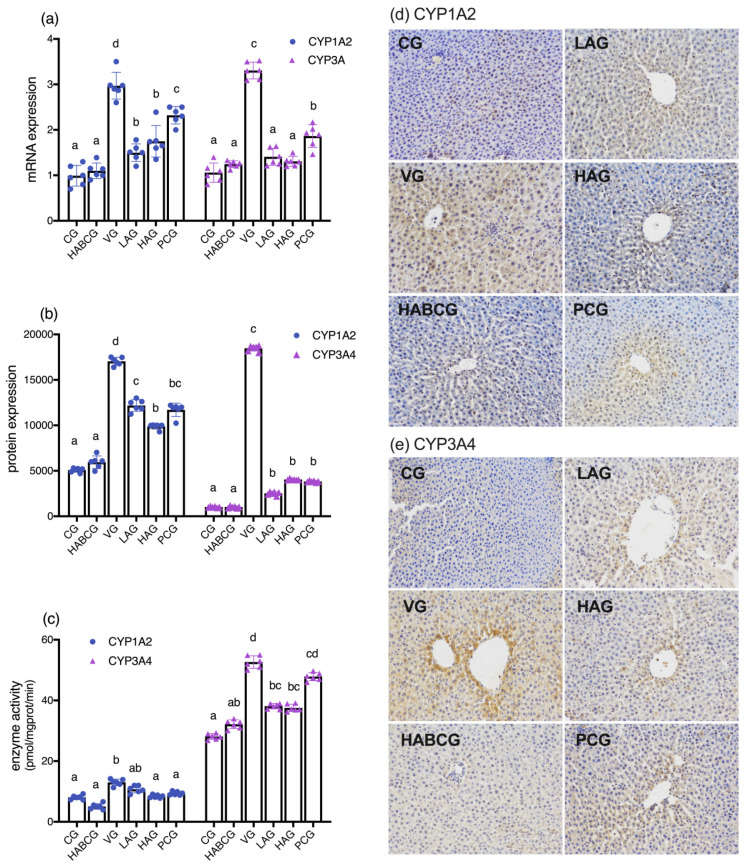
Effect of aloin supplementation on the mRNA expression of hepatic CYP1A2 and CYP3A (**a**) in rats, representative immunohistochemical photomicrographs for CYP1A2 (**d**) and CYP3A4 (**e**) of liver sections (×200), quantitative data of immunohistochemistry for CYP1A2 and CYP3A4 (**b**), and the effect of aloin supplementation on the enzyme activities of hepatic CYP1A2 and CYP3A4 (**c**) in rats. CG, control group; HABCG, high-dose aloin blank control group; VG, vehicle group; LAG, HAG, low- and high-dose aloin intervention group; PCG, positive control group; CYP1A2/3A4: cytochrome P450 1A2/3A4 enzyme (values with different letters represent statistical significance difference among groups, *p* < 0.05).

In order to evaluate the role of aloin in maintaining the antioxidant status of the liver, we detected the contents of GSH (Figure 3b), SOD (Figure 3c), CAT (Figure 3d), and GSH-PX (Figure 3e) in the liver. Compared with the control group, the levels of four antioxidants in the vehicle group showed a significant decrease (*p* < 0.05), which could explain the reason for the increase in lipid peroxidation to a certain extent. Compared with the vehicle group, the intervention of aloin increased the four antioxidant levels in the liver (*p* < 0.05), and all of them increased to the normal level. In order to explore whether the antagonism of aloin against hepatotoxicity of AFB1 was related to its effect on the GST activity of phase II enzyme, the GST level in each group was measured in the experiment (Figure 4f). It can be seen that, compared with the control group, the GST enzyme activity in the vehicle group was decreased (*p* < 0.01) after continuous intake of low-dose AFB1 for five weeks, and its level was only 57.3% of the normal value. However, the long-term intervention of aloin effectively alleviated this change. Under the condition of low-dose aloin, the GST enzyme activity was significantly increased (*p* < 0.05), which was 1.6 times as high as that in the vehicle group and was almost the same as that in the control group.

### 2.4. Effect of Aloin on Phase I Enzymes CYP1A2 and 3A4

As shown in Figure 4a, the gene expression levels of *CYP1A2* and *CYP3A* in the vehicle group were increased abnormally after five weeks of AFB1 intake, which was significantly higher than that in control group (*p* < 0.001). Interestingly, the overexpression of these two enzymes were effectively alleviated under the intervention of aloin, lower than that of the vehicle group (*p* < 0.001), in which the expression of *CYP3A* decreased to the level of the control group, indicating that aloin also played an effective down-regulation role in the expression of the phase I activating enzyme in subchronic AFB1 liver injury model.

In order to further study the effect of aloin on the AFB1 metabolic activating enzyme, the protein expression of CYP1A2 and CYP3A4 was observed by immunohistochemical staining. The observation results were shown in Figure 4d,e, and the quantitative data results were statistically shown in Figure 4b. From the immunohistochemical results, we could see that CYP1A2 and CYP3A4 positive cells were located in the cytoplasm, and the positive cells were distributed in a diffuse or flaky form. Compared with the control group, the positive cells in the vehicle group increased and spread outward from the central portal vein, with the distribution around the central portal vein being the largest. Compared with quantitative data, the expression of CYP1A2 and CYP3A4 protein in liver tissue of vehicle group was 3.6 and 23.3 times of that of the control group, respectively, which was obviously higher than normal level (*p* < 0.001), and this changing trend was consistent with the changing trend of *CYP1A2* and *CYP3A* in gene expression. Interestingly, compared with the vehicle group, the positive cells in the aloin intervention group were reduced, and the protein expressions of CYP1A2 and 3A4 were lower than those in the vehicle group (*p* < 0.001). The above results further confirmed the downregulation effect of aloin on CYP1A2 and CYP3A4 expression at the protein level. Therefore, it is speculated that aloin can reduce the carcinogenicity and hepatotoxicity of precancerous substance AFB1 by reducing the activation, thus achieving the effect of chemical prevention.

In order to further verify the inhibitory effect of aloin on phase I enzymes CYP1A2 and CYP3A4 in rat liver tissue induced by AFB1, we detected the enzyme activities of these two enzymes in each group. As shown in Figure 4c, the activities of CYP1A2 (*p* < 0.05) and CYP3A4 (*p* < 0.001) in the vehicle group were significantly higher than those in the control group, indicating that the intake of AFB1 for five consecutive weeks stimulated the metabolic activation enzymes CYP1A2 and CYP3A4, improved their activities, accelerated the activation of AFB1, and induced severe liver damage in rats. However, compared with the vehicle group, the enzyme activities of the two enzymes in the aloin groups were reduced to different degrees; CYP1A2 was reduced by 30.4% and 41.9% (*p* < 0.05) in the low-dose aloin group and CYP3A4 was reduced by 26.1% (*p* < 0.01) and 26.2% (*p* < 0.01) in the high-dose aloin group, respectively. The above results once again confirmed the inhibitory effect of aloin on AFB1 metabolic activation enzymes CYP1A2 and CYP3A4. It is speculated that aloin may inhibit the enzyme activity by reducing the expression of CYP1A2 and 3A4 at the gene and protein levels, thus blocking the activation of precancerous substance AFB1, reducing its carcinogenicity and hepatotoxicity to achieve the effects of protecting the liver.

### 2.5. Effect of Aloin on the Expression of Inflammation-Related Factors in Liver

**Figure 5:**
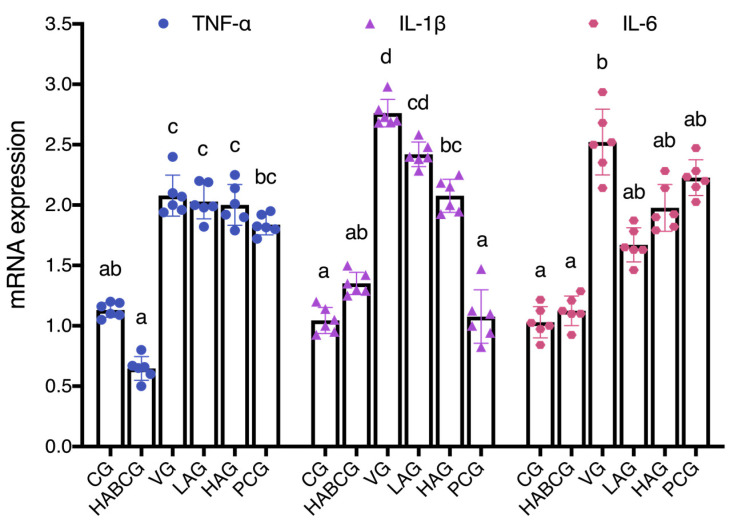
Effect of aloin supplementation on the expression of hepatic *TNF-α*, *IL-1β*, and *IL-6* in rats. CG, control group; HABCG, high-dose aloin blank control group; VG, vehicle group; LAG, HAG, low- and high-dose aloin intervention group; PCG, positive control group; TNF-α, tumor necrosis factor-α; IL–1β, interleukin-1β; IL-6, interleukin-6 (values with different letters represent statistical significance difference among groups, *p* < 0.05).

In order to further explore the role of aloin in liver inflammation induced by subchronic AFB1, we detected the expression of inflammatory cytokines *TNF-α*, *IL-1β*, and *IL-6* in liver tissues of rats in each group. The results showed (Figure 5) that compared with the vehicle group, the expression of *IL-1β* and *IL-6* in aloin groups decreased to a certain extent. The expression of *IL-1β* decreased by 6.4% and 27.2% (*p* < 0.05), and the expression of *IL-6* decreased by 34.1% and 23.9%, respectively, in the low-dose and high-dose groups, but had little effect on the expression of *TNF-α*. Therefore, the anti-inflammatory activity of aloin in the subchronic AFB1 liver injury model may be related to its down-regulation of *IL-1β* mRNA, which explains the phenomenon of aloin improving the infiltration of inflammatory cells in the liver to some extent.

## 3. Discussion

With the development of nutrition science, scientists have discovered in the study of the relationship between nutrition, health, and disease that chemical components other than the known essential nutrients in pure natural plant foods can play an important role in preventing subchronic diseases. They are called phytochemicals because they mostly come from plants, and some of them have been widely used as health food ingredients. Phytochemicals include terpenoids, organic sulfur compounds, flavonoids, and plant polysaccharides. These functional compounds play a variety of physiological functions: anti-oxidation, regulating immunity, inhibiting tumors, anti-infection, reducing cholesterol, delaying aging, and other health care functions. It is foreseeable that with the continuous deepening and development of scientific research, pure natural plant foods will play an important role in protecting human health and preventing subchronic diseases such as fatty liver, cardiovascular, and cerebrovascular diseases and cancer.

Long-term exposure to AFB1 can lead to chronic poisoning and decrease the activities of digestive tract enzymes, pancreatic lipase, trypsin, amylase, and other enzymes, resulting in nutritional absorption disorders, metabolic disorders, and anorexia, thus causing slow growth of body weight [22,23,24]. In this study, we demonstrated that aloin could relieve anorexia caused by AFB1, and its daily average food intake was significantly higher than that of the vehicle group. In the meantime, compared with the vehicle group, it is interesting to note that the slow weight growth caused by AFB1 can be effectively alleviated by intragastric administration of aloin (*p* < 0.01, Figure 1c). H&E staining of liver tissues showed that the aloin intervention group and the positive group also had varying degrees of pathological changes compared with the vehicle group. However, the degree of liver cell swelling was decreased, the structure of liver lobules was basically healthier, and inflammatory infiltration was also significantly inhibited, which indicated that aloin played a functional role in protecting liver from subchronic liver injury caused by AFB1. The staining results of liver tissue sections corresponded to the results of liver function indexes in serum. An abnormal elevation of ALT and AST contents in serum sensitively reflected liver cell damage; abnormal TBIL contents reflected hepatic and/or biliary tract abnormalities, while albumin contents can reflect the synthetic reserve function of the liver. Aloin intervention reduced ALT, AST, and TBIL levels in serum of AFB1-induced liver injury rats, which was lower than that of the vehicle group (*p* < 0.05, Figure 2b–d). Lipid peroxidation was closely related to ROS overproduction and was one of the most destructive processes in living cells. It can cause a series of physiological effects such as enzyme inactivation, osmotic fragility, and membrane fluidity changes [25]. Antioxidant status is a useful tool to evaluate the risk of oxidative damage caused by ROS, among which GSH, SOD, CAT, and GSH-PX levels are the main determinants in the body’s antioxidant defense mechanism, which can synergistically eliminate ROS and prevent lipid peroxidation [26]. From the experimental results, we could see that compared with the vehicle group, the expression level of lipid peroxidation marker MDA in aloin groups had been effectively relieved, while the contents of GSH, SOD, CAT, and GSH-PX had been significantly increased (*p* < 0.05, Figure 3b–e). At the same time, we know aloin has an excellent purgative effect and participates in many physiological functions such as antioxidation, anti-inflammation, and antivirus [27]. However, due to its significant purgative effect, the long-term use of aloin may cause damage to the intestinal tissues of the body. Therefore, this experiment explored whether the aloin dose would bring side effects to the intestinal tract. The results showed that no abnormal structure was found in the colon of rats in each group, which proved that the aloin dose used in the experiment was safe.

As a precancerous substance, AFB1 toxicity is mediated by a phase I metabolism enzyme cytochrome P450 enzyme system (CYP450). After CYP450 activates AFB1, part of AFB1 is activated by the epoxidation of cytochrome P4501A2 and 3A4(CYP1A2 and CYP3A4) to generate the final carcinogen AFB-8,9- epoxide (AFBO) [28]. AFBO is a primary high-activity electrophilic compound that mediates hepatotoxicity induced by AFB1. A part of AFBO combines with GSH to detoxify under the catalysis of GST [29,30]. Gao et al. [31] found that the inhibitory effect of phloretin on liver injury induced by AFB1 is closely related to its CYP1A2 inhibitory activity. Other studies have confirmed that the chemical intervention of lycopene on the toxicity of AFB1 is also closely related to its phase I enzyme metabolism and activation inhibition [32]. Phase II enzyme GST is the critical detoxifying enzyme of AFB1 and plays a vital role in protecting tissues from toxic effects of activated AFB1 [33,34,35]. In this experiment, we detected the mRNA expression, protein expression, and enzyme activities of CYP1A2 and CYP3A and the expression of GST level. The results confirmed the inhibitory effect of aloin on AFB1 metabolic activation enzymes CYP1A2 and CYP3A4. It is speculated that aloin may reduce its enzyme activity and increase phase II enzyme GST activity by reducing AFB1 stimulation and down-regulating its expression on gene and protein levels, thus blocking the activation of precancerous substance AFB1, reducing its carcinogenicity and hepatotoxicity to achieve the effects of protecting the liver.

More and more data showed that the increase in inflammatory cytokines is an essential factor causing liver cancer [36]. Park et al. found that aloin can inhibit inflammatory response by blocking mRNA expression of iNOS in a lipopolysaccharide-induced inflammatory model [19]. In the colitis rat model, aloin can reduce the inflammatory injury of rats by down-regulating the expression of *TNF-α* and *IL-6* [20]. In the previous acute AFB1 liver injury model, we also confirmed the anti-inflammatory activity of aloin. In order to further explore the role of aloin in liver inflammation induced by subchronic AFB1, the expression levels of inflammatory cytokines *TNF-α*, *IL-1β*, and *IL-6* in liver tissues of rats in each group were detected. Fluorescence quantitative PCR results showed that aloin blocked or delayed the occurrence and development of inflammation by down-regulating the expression of *IL-1β*, which might be another mechanism for aloin to reduce the subchronic hepatotoxicity of AFB1. Researchers found that aloin decreases the level of LPS-induced iNOS expression, inhibiting the release of IL-1β, IL-6, TNF-α, and NO dose-dependently [37]. In addition, the aloin dose dependently protects the heart against the lipid peroxidation and restore the levels of antioxidative defenses: reduce glutathione, catalase, and superoxide dismutase. It remarkably reduces the expressions of proinflammatory TNF-α, IL-1β, and IL-6, even at the lower doses [38]. Aloin may exert a potential mechanism by initiating an immune cascade and modulating inflammatory response. Therefore, the possible role of aloin in inflammation and immune response, as well as the underlying mechanisms, needs to be further elucidated.

## 4. Materials and Methods

### 4.1. Materials

Aloin, purity ≥ 98%, purchased by Chengdu Ruifensi Biotechnology Co., Ltd. (Sichaun, China). AFB1, DMSO, silymarin, 4-hydroxyethylpiperazineethanesulfonic acid (HEPES), luminol, horseradish over AFB1, DMSO, silymarin, 4-hydroxyethylpiperazineethanesulfonic acid (HEPES), BSA, Lu Mino, horseradish peroxidase, and Coomassie Brilliant Blue G-250 were purchased from Sigma (St. Louis, MO, USA). EDTA antigen repair solution and PBS (pH 7.4) buffer were purchased from Wuhan Google Biotechnology Co., Ltd. (Hubei, China). The MDA, GSH, SOD, CAT, GSH-PX, and GST kit, and hematoxylin-eosin staining kit were purchased from Nanjing Jiancheng Bioengineering Co., Ltd. (Jiangsu, China). The animal cell/tissue microsomal component separation kit and the CYP1A2 and CYP3A4 activity fluorescence quantitative detection kit were purchased from Shanghai Jiemei Gene Pharmaceutical Technology Co., Ltd. (Shanghai, China). Polyclone antibodies to CYP1A2 and CYP3A4 enzymes were purchased from Beijing Biosynthesis Biotechnology Co., Ltd. (Beijing, China). The One Drop Micro Ultraviolet Spectrophotometer was purchased from Shanghai Caiyi Biological Technology Co., Ltd. (Shanghai, China). The goat anti-rabbit/mouse universal secondary antibody and diaminobenzidine (DAB) developer were purchased from DAKO (Dako, Glostrup, Denmark). The liquid nitrogen-free RNA sample preservation solution, diethyl carbonate (DEPC), and animal total RNA extraction kit were purchased from Shanghai Jierui Biological Engineering Co., Ltd. (China). RevertAid First Strand cDNA Synthesis Kit is a product of MBI Fermentas (MBI, Burlington, ON, Canada). Power SYBR^®^ Green PCR Master Mix was purchased from ABI (Foster, CA, USA). H_2_O_2_, ammonia water, and paraformaldehyde are products of Sinopharm Chemical Reagent Co., Ltd. (Shanghai, China).

### 4.2. The Treatment of the Experimental Animal

All animal protocols used in this study were authorized by the Institutional Animal Care and Use Committee of Jiangnan University, Wuxi, China (Approval No. JN.20180417-20180510). Experimental animals are treated as humanely as possible to alleviate the pain suffered during the experiment.

The healthy male rats (5–6 weeks old) were purchased from Shanghai slack Experimental Animal Co., Ltd. After purchase, the rats were reared adaptively in the animal house for 7 days and then randomly divided into six groups (six rats in each group) according to body weight: the control group (CG), the high-dose aloin blank control group (HABCG), the vehicle group (VG), the low- and high-dose aloin intervention groups (LAG, HAG), and the positive control group (PCG). The doses of aloin intervention group were 10 and 30 mg/kg bw, respectively. HABCG was given 30 mg/kg bw aloin, and PCG was given 50 mg/kg bw silymarin. All rats were given solvent (CG and VG) or a corresponding dose of intervention by gavage once a day for 2 weeks (−14–0 days). Subsequently, rats in all groups except CG and HABCG were given 200 μg/kg bw AFB1 (dissolved in corn oil) once a day for 5 weeks (1–35 days). All rats in the AFB1 intervention group were gavaged with the corresponding dose of intervention substance (dissolved in corn oil) once a day for 5 weeks (1–35 days) after each AFB1 gavage for 1 h. CG were gavaged with corn oil of equal volume.

At the end of the experiment, the rats fasted overnight, anesthetized after weighing, and blood was quickly taken from the heart (10 mL) by ordinary vacuum blood collection vessels. After the neck was severed and executed, the abdominal cavity was quickly opened, and the liver was removed after perfusing and flushing with pre-cooled normal saline through the hepatic portal vein. After washing with normal saline, the liver was sucked dry and weighed, while the spleen, kidney, and thymus were removed and weighed. Part of liver and colon tissues were soaked in neutral formalin solution, fully fixed and used for histopathological research, liver tissue pieces of appropriate size were treated with liquid nitrogen part RNA sample preservation solution for subsequent RNA extraction, and the rest of the liver tissues were stored in an ultra-low-temperature refrigerator at −80 °C for standby after being rapidly frozen in liquid nitrogen.

### 4.3. Liver Histopathological Examination (H&E Staining)

After the liver tissue was fully fixed in formalin solution, the liver tissue structure and pathological changes were observed through an optical microscope after a series of steps of dehydration, wax dipping, paraffin embedding, slicing, fishing, baking, dewaxing, rehydration, hematoxylin staining, elution, eosin re-staining, cleaning, drying, and sealing.

### 4.4. Immunohistochemical Detection of Liver Tissue

Liver tissue was fixed in 4% neutral paraformaldehyde solution for 24–48 h, dehydrated, soaked in wax, and embedded, and then the paraffin was continuously sliced. The most polylysine slides were taken out, baked at 60 °C for 1 h, and dewaxed and rehydrated conventionally, and the slices were placed into a repair box filled with EDTA antigen repair buffer (pH 8.0), and placed into a microwave oven for antigen repair. The glass slide was cooled naturally, placed in PBS, shaken, and washed on the shaker for 5 min for three times in succession. Subsequently, the slices were placed in 3% H_2_O_2_ and treated in the dark at room temperature for 25 min to inactivate endogenous peroxidase. The slides were naturally cooled to room temperature. The slides were placed in PBS and washed three times for 5 min each time. Slices were laid flat in the box, incubated overnight at 4 °C, and washed in PBS for 5 min for three consecutive times the next day. The secondary antibody (HRP labeled by horseradish peroxidase) was added, incubated at room temperature for 50 min, and washed with PBS for 5 min each time three times in succession. Subsequently, the newly configured DAB developer was added dropwise for color development. At room temperature, the color change was observed under a light microscope to determine the optimal termination time. The slices were rinsed with distilled water, then re-dyed with hematoxylin, then conducted conventional dehydration, transparency, and natural air drying, and finally sealed with neutral gum, and the film under microscope was observed.

### 4.5. Determination of Serum Liver Function Indexes

Heart blood was collected and centrifuged at 3000 rpm for 10 min at 4 °C after clot coagulation to prepare the serum. The obtained serum was tested for total bilirubin (TBIL) content, glutamic pyruvic transaminase, glutamic oxaloacetic transaminase (ALT, AST), and alkaline phosphatase (ALP) activity by a Cobas-c501 automatic biochemical analyzer.

### 4.6. Determination of Biochemical Indexes in Liver Tissue

Livers were homogenized with saline to make a 10% homogenate, and supernatants were collected. After Coomassie brilliant blue was used to test the protein content, the levels of lipid peroxide MDA, antioxidant enzymes (SOD, CAT, and GSH-PX), non-enzymatic antioxidant GSH, and AFB1 phase II metabolic enzyme GST in liver tissue were operated according to the corresponding kit instructions, and the above indexes were corrected with the corresponding protein concentration.

### 4.7. Extraction of Total RNA and Real-Time Fluorescence Quantitative PCR

The liver tissue treated with RNA preservation solution was taken out and placed on ice after 30 min at −20 °C, and then 1 mL of pre-cooled Trizol reagent was added and fully homogenized in the ice tank with a hand-held electric homogenizer. The specific extraction operation steps were carried out according to the instructions of the total RNA extraction kit. After RNA extraction, the purity and concentration were measured by ONE DROP. According to the measured RNA concentration, the RNA was diluted to 200 ng/μL with appropriate amount of DEPC water. Oligo(dT)18 was used as a primer for reverse transcription. The obtained product was stored at −80 °C for later use. Primer 6 was used to design the primer of the target gene and the internal reference gene glyceraldehyde-3-phosphate dehydrogenase (GAPDH) according to the gene sequence, and Shanghai Jierui Biological Engineering Co., Ltd. was entrusted to synthesize the primer. The specific sequence is shown in the Supplementary Materials (Table S1). The cDNA obtained by RT-PCR was used as the template, diluted by appropriate multiples, and then real-time fluorescence quantitative PCR was carried out by SYBR Green I chimeric fluorescence method. The expression levels of the target genes were normalized to GAPDH, and the results were calculated using the 2^−ΔΔCT^ method. The purity of the PCR products was assessed by dissociation curves.

### 4.8. Preparation of Liver Microsomes

After the liver tissue was thawed on ice, three times the volume of pre-cooling homogenate buffer was removed and added. Afterwards, full homogenate, 4 °C, 9000 g, was centrifuged for 30 min. The supernatant was carefully removed and the precipitate was discarded. A total of 100,000 g of the supernatant was centrifuged at 4 °C for 60 min, and the supernatant (cytoplasmic part) was carefully removed and stored at −80 °C immediately after sub packaging. A total of 1 mL of pre-cooling cleaning buffer (do not mix evenly) was added to the obtained precipitate. A total of 100,000 g was centrifuged at 4 °C for 10 min, the supernatant was discarded, and the obtained precipitate was purified microsome.

### 4.9. Determination of Activity of Liver Phase I Drug Metabolizing

In the experiment, the enzyme activity of CYP1A2 and CYP3A4 in liver microsomes was determined by the fluorescence quantitative detection method. The specific operation was carried out according to the instructions of the CYP1A2 activity fluorescence quantitative detection kit and the CYP3A4 activity fluorescence quantitative detection kit.

### 4.10. Data Processing and Analysis

All the data in each group were expressed by the mean standard error (mean ± SEM). SPSS 18.0 software (SPSS, Inc., IBM Company, Chicago, IL, USA) was used to compare the experimental data between groups and to test the significance of differences by one-way ANOVA method and Duncan test. *p* < 0.05 is a significant difference.

## 5. Conclusions

In short, Aloin can inhibit the development process of subchronic AFB1-induced hepatotoxicity, anorexia, slow weight growth, and atrophy of immune organs. In the meantime, it effectively improves the pathological changes of rat liver tissue and reduces the degree of liver cell degeneration and inflammation induced by subchronic AFB1. Aloin suppress the generation of MDA in rat liver, effectively increases the levels of GSH, SOD, CAT, and GSH-PX, and has potent antioxidant and free radical scavenging capabilities, thus reducing liver oxidative stress damage and achieving the effect of protecting liver. It can also restrain the expression and activity of phase I enzymes CYP1A2 and CYP3A4, enhance the activity of phase II enzyme GST, reduce the metabolism of precancer, and increase the detoxification of final carcinogen, thus reducing its hepatotoxicity and achieving the liver-protecting effect. Furthermore, aloin can block or delay the occurrence and development of inflammation by down-regulating *IL-1β* expression, which may be another mechanism of aloin in reducing the subchronic hepatotoxicity of AFB1.

## Data Availability

Not applicable.

## Supplementary Materials

The following are available online at https://www.mdpi.com/article/10.3390/ijms222111620/s1.
