# Supplementary material for: The Intervention and Mechanism of Action for Aloin against Subchronic Aflatoxin B1 Induced Hepatic Injury in Rats
Source: Int J Mol Sci. 2021 Oct 27;22(21):11620. doi: 10.3390/ijms222111620 (PMC8584096; doi:10.3390/ijms222111620)
Supplement: Supplementary file 1 [file ijms-22-11620-s001.zip › ijms-1401217-supplementary.pdf]

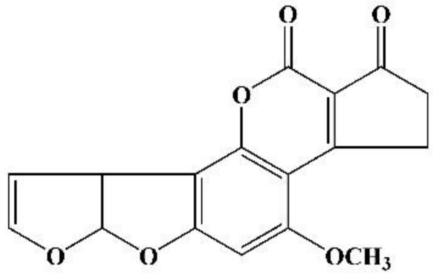

AFB_1

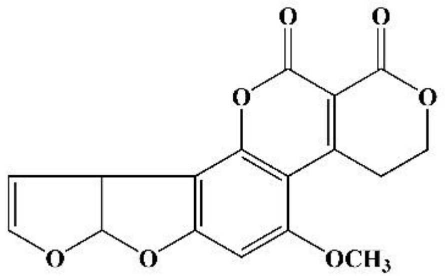

AFG_1

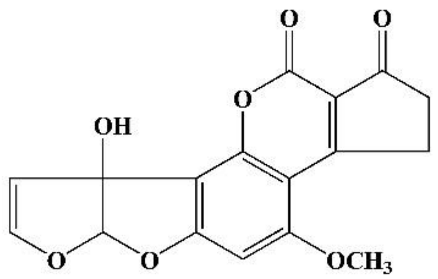

AFM_1

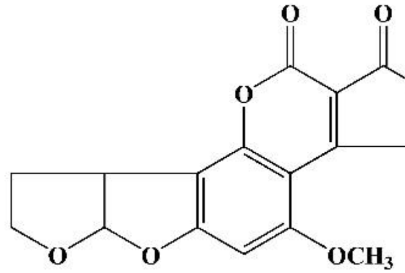

AFB_2

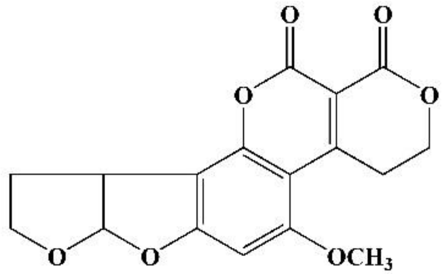

AFG_2

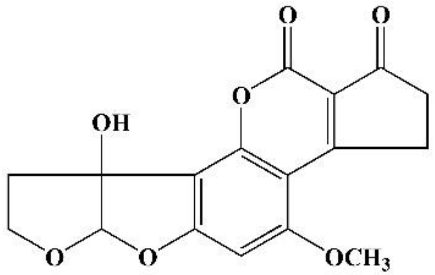

AFM_2

Figure S1. Chemical structure of the key aflatoxins

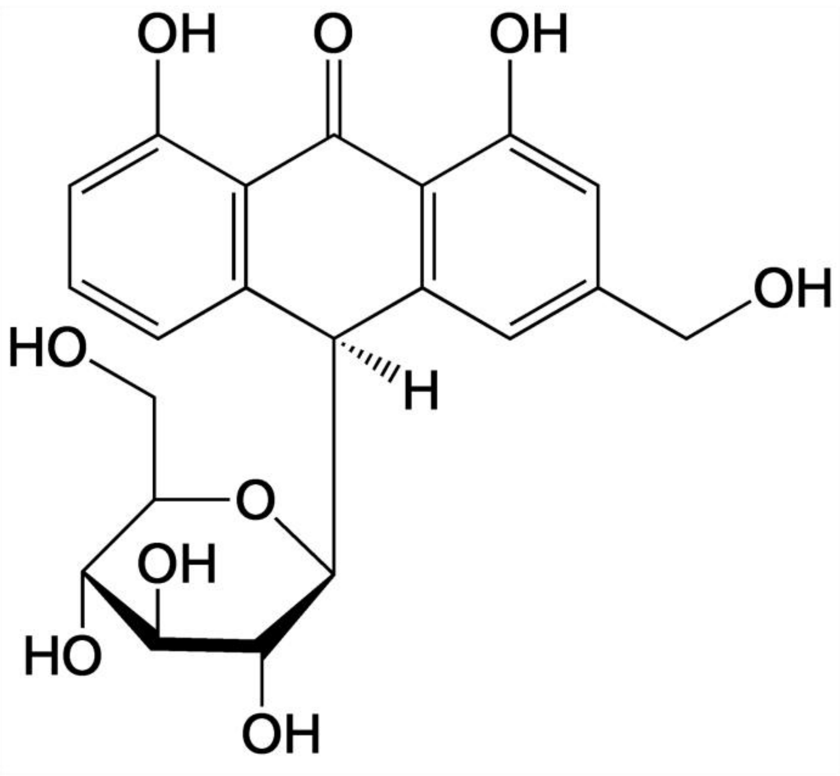

Figure S2. Chemical structure of aloin

Table S1. The specific primer sequence information

Name	Sequences of primers (5′–3′)
GADPH	Sense: 5'- CAACGGGAAACCCATCACCA-3'<br>Antisense: 5'- ACGCCAGTAGACTCCACGACAT-3'
CYP1A2	Sense: 5'- CCCTGAAGAGTTTCTCCATAGCC-3'<br>Antisense: 5'- GATGACATTAGCCACCGATTCC-3'
CYP3A	Sense: 5'- GGCAAACCTGTCCCTGTGAAAG-3'<br>Antisense: 5'- TGGCGTGAGGAATGGAAAGAGTA-3'
IL-1β	Sense：5'-GTGGTATTCTCCATGAGCTTTGTA-3'<br>Antisense：5'-CCATCTTCTTCTTTGGGTATTGTT-3'
TNF-α	Sense：5'-TCTGTCTACTGAACTTCGGGGTGAT-3'<br>Antisense：5'-CTGCTTGGTGGTTTGCTACGAC-3'
IL-6	Sense：5'- AGACTTCACAGAGGATACCACCCAC-3'<br>Antisense: 5'- CAATCAGAATTGCCATTGCACAA-3'
